# Upregulated TNF Expression 1 Year After Bariatric Surgery Reflects a Cachexia-Like State in Subcutaneous Adipose Tissue

**DOI:** 10.1007/s11695-016-2477-5

**Published:** 2016-11-29

**Authors:** Alexander Jürets, Bianca Karla Itariu, Magdalena Keindl, Gerhard Prager, Felix Langer, Viktor Grablowitz, Maximilian Zeyda, Thomas Michael Stulnig

**Affiliations:** 1 Christian Doppler Laboratory for Cardio-Metabolic Immunotherapy and Clinical Division of Endocrinology and Metabolism, Department of Medicine III, Medical University of Vienna, Waehringer Guertel, 18-201090 Vienna, Austria; 2 Department of Surgery, Medical University of Vienna, Vienna, Austria; 3 Department of Surgery, Göttlicher Heiland Hospital, Vienna, Austria; 4 Department of Pediatrics and Adolescent Medicine, Clinical Division of Pediatric Pulmonology, Allergology and Endocrinology, Medical University of Vienna, Vienna, Austria

**Keywords:** Tumor necrosis factor, Bariatric surgery, Subcutaneous adipose tissue, Gene expression, Weight loss

## Abstract

**Background:**

Adipose tissue dysfunction contributes to obesity-associated chronic diseases. In the first year after bariatric surgery, obese patients significantly improve their metabolic status upon losing weight. We aimed to investigate whether changes in subcutaneous adipose tissue gene expression reflect a restoration of a healthy lean phenotype after bariatric surgery.

**Methods:**

Thirty-one severely obese patients (BMI ≥ 40 kg/m^2^) were examined before and after surgery. subcutaneous adipose tissue (SAT) was collected during and 1 year after bariatric surgery. SAT from 20 matched lean and overweight patients (BMI < 30 kg/m^2^) was collected during elective abdominal surgery. Baseline characteristics and SAT gene expression relevant to glucose and lipid metabolism, inflammation, and apoptosis were analyzed.

**Results:**

After surgery, mean BMI decreased from 46.1 ± 6.3 to 31.1 ± 5.7 kg/m^2^ and homeostasis model assessment of insulin resistance from 5.4 ± 5.3 to 0.8 ± 0.8. SAT expression of most analyzed inflammatory cytokines, growth factors, and metabolic and cell surface markers was greatly downregulated even compared to the lean cohort. In contrast, gene expression of *TNF* and *CASP*3 was significantly upregulated. Elastic net regression analysis showed that fasting glucose levels and *CASP3* predicted increased *TNF* expression in the post-obese group.

**Conclusions:**

Gene expression patterns in SAT 1 year after bariatric surgery point to a reduced inflammation. The unexpected high TNF expression in SAT of post-obese subjects is most likely not an indicator for inflammation, but rather an indicator for increased lipolysis and adipose tissue catabolism. Notably, after bariatric surgery SAT gene expression reflects a cachexia-like phenotype and differs from the lean state.

**Electronic supplementary material:**

The online version of this article (doi:10.1007/s11695-016-2477-5) contains supplementary material, which is available to authorized users.

## Introduction

Adipose tissue participates in whole-body metabolism not just by energy storage. It is an endocrine organ and an important site for inflammatory crosstalk and immune response. Subcutaneous adipose tissue (SAT) is the largest adipose compartment for most humans and the primary site for lipid storage. Excess caloric intake per se causes an activation of resident immune cells in the adipose tissue, to which other immune cells are attracted from the circulation [1]. Long-term overnutrition leads to a chronic low-grade inflammation, which promotes insulin resistance, for instance via insulin receptor substrate 2 (*IRS2*) downregulation in SAT [2] and thus precedes type 2 diabetes mellitus (T2DM) and other metabolic disorders [3]. In the development of chronic inflammation, T cells induce the infiltration and inflammatory polarization of macrophages in the adipose tissue [4, 5], which can make up to 40 % of cell content in obesity. These inflammatory macrophages have higher lipid content in murine models of obesity and show increased expression of genes relevant to lipid metabolism, such as peroxisome proliferator nuclear receptor gamma (*PPARG*) [6]. Additionally, immune cells are the major source of several inflammatory cytokines such as the cachexia-inducing tumor necrosis factor (*TNF*)-α, interleukin 6 (*IL6*), and interleukin 1β (*IL1B*) [12]. Notably, insulin resistance and lipolysis are both mediated by TNF. Lipolysis is enhanced by TNF through downregulating of perilipin 1 (*PLIN1*), which protects adipocytes from lipolysis by modulating substrate availability for hormone-sensitive lipase [7], and cell death-inducing DNA fragmentation factor-alpha-like effector A (*CIDEA*) expression, which is enriched at lipid droplet contact sites [8, 9]. SAT Expression of both *PLIN1* and *CIDEA* correlates with insulin sensitivity in obesity [9]. Moreover, adipose tissue of obese compared to lean subjects secretes reduced concentrations of the insulin-sensitizing adipokine adiponectin (*ADIPOQ*) [10]. Overall, these cytokines and adipokines play a crucial role in the development of adipose tissue inflammation and insulin resistance [11–13].

Weight loss is key to restoring insulin sensitivity and reducing cardiovascular risk. Currently, bariatric surgery is the most sustainable treatment option, superior to lifestyle interventions in terms of weight maintenance and risk reduction [14]. Long-term remission of T2DM in obese patients undergoing bariatric surgery has been frequently reported: 1 year after surgery, the hemoglobin A_1c_ level improved significantly, and disease-related mortality was reduced compared to conservative therapy [15, 16]. On the other hand, weight loss following bariatric surgery resembles in some cases of cachexia [17]. Although cachexia and obesity are on opposite sides of the weight spectrum, they display many similarities as both are characterized by increased adipose tissue lipolysis and insulin resistance.

Growth factors, which are important for adipogenesis, are downregulated in catabolic states and cachexia, which are characterized by low levels of insulin growth factor 1 (IGF-1) and increased levels of transforming growth factor beta (TGF-β) family members [18]. Myostatin is a member of the TGF-β family and a negative regulator of muscle growth; however, it is not clear how weight loss after bariatric surgery affects serum myostatin levels.

Changes in adipose tissue gene expression during weight loss after bariatric surgery treatment of morbid obesity are to date underinvestigated. We set out to investigate the effects of weight loss on genes relevant to inflammation, lipolysis, apoptosis, and growth in adipose tissue 1 year after surgery and to compare expression levels with an age- and sex-matched lean and overweight control group.

## Material and Methods

### Ethical Statement

The trial has been performed in accordance to the 1964 Declaration of Helsinki and its latter amendments and with the Good Clinical Practice guidelines at the Department of Medicine III, Medical University of Vienna. All participants provided informed written consent prior to their inclusion in the study. The trial had been approved by the Ethics Committee of the Medical University of Vienna (EK-Nr. 488/2006, EK 275/2006, and EK-Nr. 963/2009) and by the Ethics Committee of the Göttlicher Heiland Hospital (EK-Nr. E10-N01-01) and was conducted at the Clinical Research Unit of the Division of Endocrinology and Metabolism, Department of Medicine III, Medical University of Vienna.

### Study Population

Morbidly obese patients (BMI > 40 kg/m^2^) prior to and 1 year after bariatric surgery were compared to a group of lean and overweight patients. We investigated metabolic parameters and SAT gene expression of 31 morbidly obese patients (BMI > 40 kg/m^2^) before and approximately 1 year after elective bariatric surgery (follow-up time 419 ± 58 days). Adipose tissue and serum samples were collected within another study from *n* = 20 lean and overweight donors (BMI < 30 kg/m^2^) undergoing elective abdominal surgery either at the Department of Surgery from the Medical University of Vienna or the Hospital Göttlicher Heiland in Vienna. Results have been published elsewhere [19, 20]. For comparison of gene expression, a subgroup of *n* = 20 obese patients matched for age and sex with the lean and overweight were analyzed.

Obese patients were advised to maintain usual physical activity at a constant level. No intensive exercise routines were documented. All obese patients participated in previous trials by our group and metabolic changes 1 year after bariatric surgery have been presented elsewhere [21]. Briefly, 20–65-year-old patients scheduled to undergo bariatric surgery or elective abdominal surgery were eligible for inclusion if their BMI was >40 kg/m^2^ before surgery for the obese group and <30 kg/m^2^ for the lean and overweight control group, and their fasting plasma glucose <126 mg/dl and 2-h plasma glucose after a 75-g oral-glucose tolerance test <200 mg/dl. Exclusion criteria included acute illness within the past 14 days or severe chronic illness such as active malignancies; acquired immunodeficiency (HIV infection, AIDS); significant liver, cardiovascular, renal, pulmonary or thyroid disease; anemia; inborn or acquired bleeding disorders; as well as pregnancy or breastfeeding. Anthropometric parameters were determined at inclusion and at the 1-year follow-up visit after bariatric surgery. All patients were Caucasian.

### Collection of Subcutaneous Adipose Tissue

All bariatric surgeries (26 Roux-en-Y-gastric bypass (RYGB), three sleeve resections, and two gastric bandings) had been performed at the Department of Surgery of the Medical University of Vienna. Samples of SAT were collected by excision from the umbilical area during surgery. Other elective abdominal surgeries had been performed at the Department of Surgery of the Medical University of Vienna and the Göttlicher Heiland Hospital, Vienna. SAT was collected by excision from the periumbilical region. One year after bariatric surgery, the post-obese SAT was collected by needle biopsy of the periumbilical abdominal area. All adipose tissue samples were immediately immersed in RNAlater (Life Technologies) and stored at −80 °C.

### Serum Samples and Laboratory Analysis

Blood samples were collected by venous puncture after a 12-h overnight fast. Blood samples were analyzed by the Department of Laboratory Medicine, Medical University of Vienna, for routine analysis of triglycerides, total-, HDL- and LDL-cholesterol, alanine transaminase (ALT), gamma-glutamyl transferase (GGT), glucose, insulin, C-peptide, C-reactive protein (CRP), glycosylated hemoglobin (HbA_1c_), and red and white blood cell counts. For ELISA analyses, blood was centrifuged at 3000*g* for 10 min at 4 °C and then stored at −20 °C. We used commercial ELISA kits to measure plasma concentrations of IL-6 (R&D Systems, Minneapolis, MN, USA) and serum concentrations of high-sensitivity tumor necrosis factor (Invitrogen, Thermo Fisher Scientific, Waltham, MA, USA) and myostatin (R&D Systems, Minneapolis, MN, USA) and a RIA for measuring serum adiponectin concentrations (Merck Millipore, Billerica, MA, USA). Homeostasis model assessment of insulin resistance (HOMA-IR) was calculated as the product of fasting insulin (μU/ml) and glucose (mg/dl) divided by 405.

### Adipose Tissue RNA Extraction and Gene Expression Analysis

Total RNA from tissue was extracted by using the RNeasy Lipid Tissue Mini Kit (QIAGEN, Venlo, Netherlands) and by TRIzol reagent (Thermo Fisher Scientific, Waltham, MA, USA) with a tissue homogenizer, followed by RNA isolation according the manufacturer’s instructions. RT-PCR was performed in duplicates by using the TaqMan Gene Expression Assays (Applied Biosystems). Gene expression was analyzed according to the ddCt method relating gene of interest Ct values to ubiquitin C (*UBC*). No significant differences in the Ct values of UBC were observed between the groups. To ensure that SAT biopsies are not contaminated with dermis tissue, keratin 18 (*KRT18*) expression was analyzed and three positive post-obese samples (gene expression >1000 % post-obese sample in comparison to the obese sample of the same patient) were excluded. A list of used TaqMan probes is provided in the supplementary Table 1.

### Statistical Analysis

Normal distribution was determined by the Shapiro-Wilk test of normality. Normally distributed data were presented as means ± SDs, otherwise as medians (interquartile ranges). Group differences were calculated by one-way ANOVA with Tukey post hoc test or Kruskal-Wallis rank sum with Dunn’s multiple comparison test, and paired *t* test, or Wilcoxon signed rank test for paired samples, as appropriate.

For gene expression comparison ddCt-data were investigated independently by linear mixed effects analysis of gene expression, with the fixed effects status (lean, obese, or post-obese) and plate (obese before and after bariatric surgery or lean and obese) and the patient ID as a random effect. No apparent deviations from homoscedasticity or normality were observed in the residual plot. Relative gene expression boxplots were normalized to the median of the obese group. Data were not adjusted for multiple testing due to the study’s exploratory nature. For Pearson’s *r* correlation, nonparametric data were log-transformed. For the elastic net equation, the optimal settings for *α* (between 0 and 1) and for *λ* plus one standard error [22] were chosen to minimize the mean squared error using leave-one-out cross-validation. To investigate the relationship of TNF with other genes, we correlated its expression with assessed parameters in the post-obese group and performed an unbiased machine learning analysis via elastic net regularization, to check for independent predictors.

For statistical analysis, we used RStudio (version 0.99.491) [23] with R (version 3.2.2) [24] with the *lme4* (1.1–10) [25] and the *glmnet* (2.0–2) packages [26]. Differences were considered statistically significant at two-sided values of *p* < 0.05.

## Results

### Changes in Anthropometric Measurements and Metabolic Parameters after Bariatric Surgery

Obese patients were matched to lean patients according to age and sex. Anthropometric measurements and metabolic parameters of the obese group at baseline and 1 year after bariatric surgery (post-obese) compared to the lean group are reported in Table 1. Post-obese patients significantly lost weight (mean ΔBMI 15 kg/m^2^) and showed improved metabolic parameters such as serum lipids (triglycerides, total cholesterol LDL-C); liver tests (ALT, GGT); and CRP concentration. Changes in glucose metabolism including insulin sensitivity and pancreatic β cell function, assessed by HOMA-IR and other OGTT-derived indices were extensive and have already been presented elsewhere [21].

**Table 1:** Characteristics of lean, obese, and post-obese patients

	Lean (*n* = 20)	Obese (*n* = 31)	Post-obese (*n* = 31)	*p* value
Sex (f/m)	15/5	24/7	24/7	
Age (years)	43 ± 9	42 ± 12	43 ± 12	0.89
Anthropometric measurements				
BMI (kg/m^2^)	25.5 ± 3.1*	46.1 ± 6.3^†^	31.1 ± 5.7^‡^	<0.01
WHR	0.90 (0.84–0.94)	0.91 (0.87–0.95)	0.88 (0.85–0.92)	0.22
Metabolic parameters				
Triglycerides (mg/dl)	125.0 (95.0–180.5)	138.0 (120.5–193.5)^†^	94.0 (71.5–116.5)^‡^	<0.01
Total cholesterol (mg/dl)	181.5 ± 34.9*	205.0 ± 33.0^†^	165.5 ± 32.3	<0.01
HDL-C (mg/dl)	38.0 (35.3–41.8)*	45.0 (41.0–53.0)^†^	51.0 (45.5–55.5)^‡^	<0.01
LDL-C (mg/dl)	116.3 (90.7–124.1)*	134.0 (102.5–140.0)^†^	90.8 (81.4–104.1)^‡^	<0.01
ALT (U/l)	14.0 (10.5–24.5)*	27.0 (21.0–35.5)^†^	19.5 (15.3–24.8)^‡^	<0.01
GGT (U/l)	23.0 (16.0–38.0)*	39.0 (22.0–50.0)^†^	16.0 (11.0–27.0)^#^	<0.01
Fasting glucose (mg/dl)	95.6 ± 15.9	94.4 ± 9.8^†^	77.8 ± 8.1^‡^	<0.01
Insulin (μU/ml)	8.0 (2.5–15.6)*	17.1 (6.2–30.8)^†^	2.0 (2.0–4.0)^‡^	<0.01
C-peptide (ng/ml)	0.5 (0.1–0.8)*	3.9 (2.9–4.7)^†^	1.9 (1.4–2.3)^‡^	<0.01
HOMA-IR	2.0 (0.5–3.2)*	4.0 (1.4–7.8)^†^	0.4 (0.4–0.9)^‡^	<0.01
CRP (mg/dl)	0.10 (0.08–0.23)*	0.89 (0.36–1.16)^†^	0.2 (0.1–0.3)	<0.01
Myostatin (ng/ml)	2.7 (2.1–3.4)*	3.8 (2.5–4.5)^†^	2.9 (2.3–3.6)	0.03
HbA1c (%)		5.5 ± 0.4	5.3 ± 0.3	<0.01
IL-6 (pg/ml)		3.2 (2.3–4.6)	1.4 (1.0–1.7)	<0.01
Adiponectin (μg/ml)		7.4 ± 2.8	11.2 ± 5.0	<0.01
TNF (pg/ml)		2.7 ± 0.9	1.8 ± 0.6	<0.01
IGF1 (ng/ml)		108.0 (95.0–125.5)	127.0 (85.8–188.3)	0.06
Albumin (mg/dl)		41.7 ± 2.4	41.2 ± 2.8	0.22

Values are depicted as mean ± SD or median (IQR). Statistical significance was calculated by one-way ANOVA with Tukey post hoc test or Kruskal-Wallis rank sum with Dunn’s multiple comparison test as appropriate and for HbA1c, IL-6, adiponectin, and TNF by paired *t* test or Wilcoxon signed rank test for paired samples

*ALT* alanine transaminase, *CRP* C-reactive protein, *GGT* gamma-glutamyl-transferase, *HbA1c* hemoglobin A1c, *HDL-C* high-density lipoprotein cholesterol, *HOMA-IR* homeostatic model assessment–insulin resistance, *LDL-C* low-density lipoprotein cholesterol, *WHR* waist to hip ratio

*Lean vs obese = *p* < 0.05

^†^Obese vs post-obese = *p* < 0.05

^‡^Lean vs post-obese = *p* < 0.05

^#^Lean vs post-obese = *p* = 0.52

Myostatin serum levels were significantly increased in obese subjects compared to lean but normalized after weight loss (Table 1). After surgery plasma IL-6 and TNF concentrations decreased significantly, whereas adiponectin concentration increased. Although the BMI of the post-obese group was higher compared to the lean group, some metabolic parameters including plasma triglycerides, fasting glucose, insulin, and HOMA-IR were significantly lower (Table 1).

### SAT Expression of Inflammatory Cytokines

**Fig. 1:**
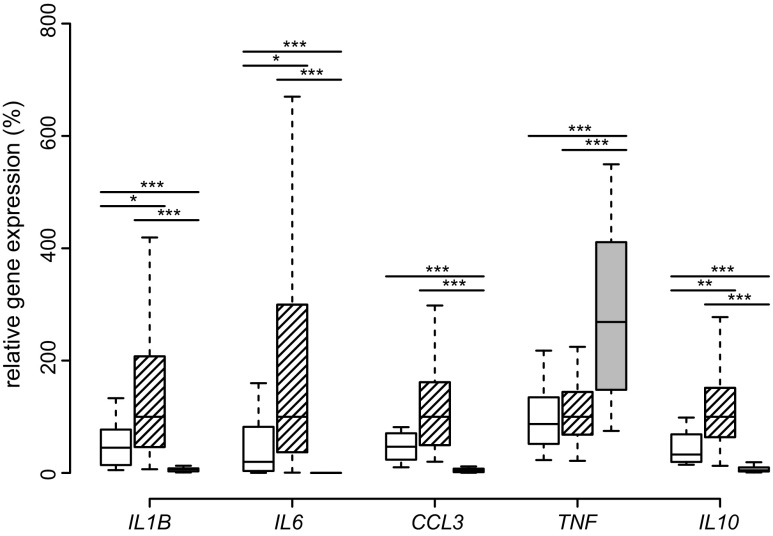
SAT expression of inflammatory cytokines. Boxplots of *IL1B*, *IL6*, *CCL3*, *TNF*, and *IL10* expression in SAT of lean (*white*), obese (*hatched*), and post-obese (*gray*) subjects are presented. Comparisons were made between lean-obese, obese-post-obese, and post-obese-lean subjects by Tukey’s Test. *p < 0.05; **p < 0.01; ***p < 0.001

Differences in SAT gene expression in the obese, post-obese, and lean group were analyzed by linear mixed effects model. In comparison, most inflammatory genes (*IL1B*, *IL6*, *CCL3*) in the post-obese group were dramatically downregulated. Notably, the cachexia-associated, pro-inflammatory *TNF* was upregulated 2.9-fold after weight loss, while expression of all interleukins (*IL1B, IL6, IL10*), as well as *CCL3* was much lower than in the lean group (Fig. 1).

### SAT Expression of Relevant Metabolic Genes

**Fig. 2:**
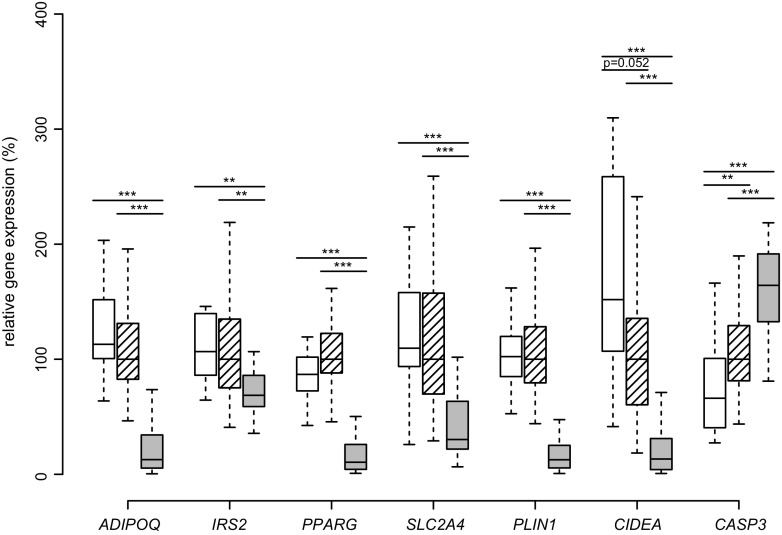
SAT expression of relevant metabolic genes. Boxplots of *ADIPOQ*, *IRS2*, *PPARG*, *SCL2A4*, *PLIN1*, *CIDEA*, and *CASP3* expression in SAT of lean (*white*), obese (*hatched*), and post-obese (*gray*) subjects are presented. Comparisons were made between lean-obese, obese-post-obese, and post-obese-lean subjects by Tukey’s test. *p < 0.05; **p < 0.01; ***p < 0.001

Since inflammatory gene expression, except TNF, was greatly reduced after surgery, we investigated the impact of weight loss on relevant metabolic genes. Compared only with the lean group, SAT *ADIPOQ* expression of obese patients before surgery was in trend lower (*p* = 0.07). In the post-obese group, *ADIPOQ* expression was significantly downregulated after weight loss. We detected a similar expression pattern between the SAT expression of metabolism-relevant genes such as *IRS2*, *PPARG*, and *SLC2A4*: while lean and obese group did not differ, messenger RNA (mRNA) expressions of the post-obese group compared to the lean and the obese groups were dramatically reduced (*p* < 0.01, Fig. 2). SAT expression of regulators of lipid turnover showed that mRNA expression of the antilipolytic lipid droplet proteins *PLIN1* and *CIDEA* are significantly downregulated in the post-obese group. In contrast, the apoptosis effector gene caspase-3 (*CASP3*) is upregulated in obese and even further upregulated in post-obese patients (Fig. 2).

### SAT Expression of Cell Surface Molecules

**Fig. 3:**
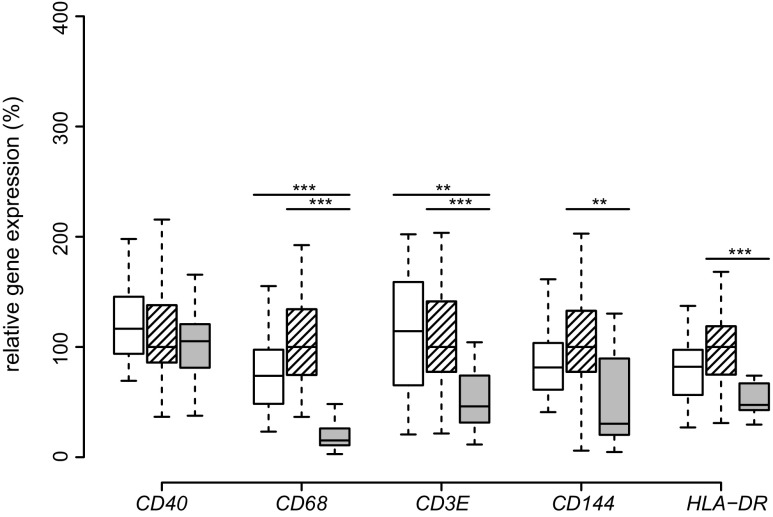
SAT expression of cell surface molecules. Boxplots of *CD40*, *CD68*, *CD3E*, *CD144*, and *HLA-DR* expression in SAT of lean (*white*), obese (*hatched*), and post-obese (*gray*) subjects are presented. Comparisons were made between lean-obese, obese-post-obese, and post-obese-lean subjects by Tukey’s test. *p < 0.05; **p < 0.01; ***p < 0.001

Since the expression of *CASP3* was upregulated in response to weight loss, we investigated whether the expression of cell surface marker would reflect the catabolic processes induced by apoptosis. The M1 macrophage marker *CD40* did not differ in mRNA expression between the groups and seemed unaffected by weight loss. In contrast, SAT expression of markers for macrophages (*CD68*), T cells (*CD3E*), endothelial cells (*CD144*), and antigen-presenting cells (*HLA-DR*) decreased significantly compared to their expression before surgery. Of note, in the post-obese group SAT expression of T cell marker *CD3E* and of macrophage marker CD68 was even more reduced compared to the lean group (Fig. 3).

### SAT Expression of Growth Factors

**Fig. 4:**
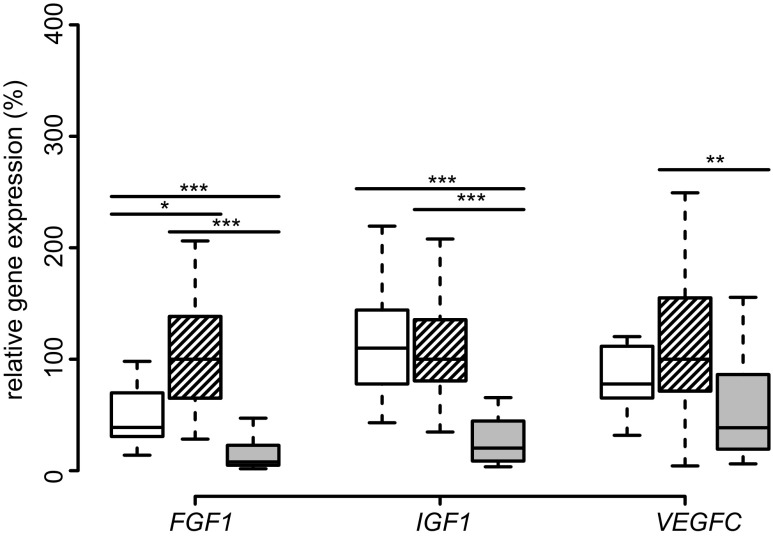
SAT expression of growth factors. Boxplots of *FGF1*, *IGF1*, *VEGFC* expression in SAT of lean (*white*), obese (*hatched*), and post-obese (*gray*) subjects are presented. Comparisons were made between lean-obese, obese-post-obese, and post-obese-lean subjects by Tukey’s test. *p < 0.05; **p < 0.01; ***p < 0.001

We next analyzed the SAT expression of growth factors relevant to obesity. The fibroblast growth factor 1 (*FGF1*), insulin growth factor 1 (*IGF-1*), and angiogenic vascular endothelial growth factor C (*VEGFC*) were downregulated after bariatric intervention (Fig. 4). Expression of *FGF1* and *IGF1* was even below the values of the lean group.

### Correlation Analysis of TNF Gene Expression after Bariatric Surgery

One of the most noteworthy findings in the post-obese group was that *TNF* expression was markedly upregulated upon weight loss (Fig. 1). Therefore, we investigated factors modulating *TNF* expression in serum or on gene expression level. By calculating Pearson’s correlation coefficient, we observed a positive correlation between *TNF* expression, fasting blood glucose, and *CASP3* expression and a negative correlation with *IGF1*, *PLIN1*, *PPARG*, and *SLC2A4* expression (Table 2). To identify independent predictors of *TNF* expression, we performed a linear regression analysis with an elastic net regularization, in which all parameters were included. The analysis showed that fasting glucose levels (mg/dl), *CASP3*, and *CD40* increase, whereas *SLC2A4* decreases *TNF* expression in the post-obese group.

**Table 2:** Correlations and elastic net regression for *TNF* gene expression in post-obese SAT

	*r*	*p* value		*r*	*p* value
Age (years)	0.02	0.93	Gene expression		
BMI (kg/m^2^)	0.01	0.95	ADIPOQ	−0.38	0.06
WHR	0.02	0.94	*CASP3*	*0.56*	*<0.01*
			CCL3	−0.34	0.18
Serum parameters			CD144	−0.29	0.14
Triglycerides (mg/dl)	0.20	0.33	CD3E	0.30	0.13
Total cholesterol (mg/dl)	−0.23	0.25	CD40	0.34	0.09
HDL-C (mg/dl)	−0.26	0.20	CD68	−0.29	0.15
LDL-C (mg/dl)	−0.25	0.21	CIDEA	-0.37	0.06
ALT (U/l)	0.23	0.21	FGF1	−0.33	0.10
GGT (U/l)	0.31	0.13	HLA-DR	0.10	0.63
*Glucose* (*mg*/*dl*)	*0.39*	*0.04*	*IGF1*	*−0.43*	*0.03*
Insulin (μU/ml)	0.00	0.98	IL10	−0.18	0.40
C-peptide (ng/ml)	−0.03	0.87	IL1B	−0.17	0.42
HOMA-IR	0.02	0.91	IL6	−0.33	0.13
CRP (mg/dl)	0.12	0.56	IRS2	−0.15	0.47
HbA1c (%)	−0.03	0.90	*PLIN1*	*−0.44*	*0.03*
Myostatin (ng/ml)	0.22	0.37	*PPARG*	*−0.48*	*0.01*
IL-6 (pg/ml)	0.26	0.20	*SLC2A4*	*−0.54*	<*0.01*
Adiponectin (μg/ml)	0.03	0.90	VEGFC	−0.27	0.18
TNF (pg/ml)	0.07	0.72			
IGF1 (ng/ml)	−0.25	0.20			
Albumin (mg/dl)	0.24	0.21			
Elastic net regression			Selected variables	*B* coefficients	
			Glucose (mg/ml)	0.002	
			CASP3 (a.u.)	0.308	
			CD40 (a.u.)	0.033	
			SLC2A4 (a.u.)	−0.85	

Significant correlations are highlighted in italics. *B* coefficients represent the unstandardized regression coefficient of the independent variables, which describe the ddCt values of TNF via elastic net regression. Elastic net parameters were chosen to minimize the mean squared error of the linear regression as following: the relative strength of the L1 and L2 penalty term *α* = 0.86 and the regression penalty *λ* = 0.30. The intercept of the regression was 0.950

*r* Pearson correlation coefficient, *p* significance level, *ALT* alanine transaminase, *CRP* C-reactive protein, *GGT* gamma-glutamyl-transferase, *HbA1c* hemoglobin A1c, *HDL-C* high-density lipoprotein cholesterol, *HOMA-IR* homeostatic model assessment–insulin resistance, *LDL-C* low-density lipoprotein cholesterol, *WHR* waist to hip ratio, *a.u.* arbitrary units

## Discussion

Our present work aimed to investigate SAT expression of relevant inflammatory and metabolic genes of morbidly obese patients before and 1 year after bariatric surgery in comparison to lean subjects. Even though the mean BMI of patients in the post-obese group was still higher compared to the lean control group, most metabolic risk factors, such as insulin resistance, hyperlipidemia, and systemic inflammation, changed to such an extent that many of the analyzed parameters in serum differed even from the lean control group. Interestingly, analysis of adipose tissue inflammation-associated genes showed that gene expression profile of the post-obese group was significantly different in comparison to the obese and even the lean control group and pointed to reduced inflammation. We further showed that weight loss affected several genes relevant to lipolysis, apoptosis, and also growth factors with a role in cachexia. Accordingly, it seems that the near to normal weight achieved after bariatric surgery reflects a cachexia-like state rather than a healthy lean phenotype, particularly indicated by the extensive upregulation of TNF expression in the post-obese group.

Macrophage-associated inflammatory cytokines like *IL1B*, *IL6*, and *CCL3* were all upregulated in obese SAT [27–29], but markedly downregulated after bariatric surgery, concordant with other data [30, 31]. Interestingly, the anti-inflammatory *IL10* was downregulated upon weight loss. The finding that TNF was markedly upregulated contrasts other data, however, in those studies, the BMI change in response to weight loss was lower, the follow-up time was shorter, or the cohorts included diabetic-patients [30, 32].

TNF was originally described as the cachexia-inducing factor and termed cachectin indicating a Janus-type function [33]. It is known to mediate lipolysis and insulin resistance by downregulation of *PLIN1* [7] and *CIDEA* expression [8]. TNF was described to cause apoptosis of adipocytes in vitro in a *CASP3*-dependent manner [34]. Notably, *CASP3* expression is higher in SAT in obesity in our study as well as in other studies [35]. Since the number of adipocytes in adults is described to be unchanged upon weight gain or weight loss [36], *CASP3* upregulation might be explained by increased apoptosis of inflammatory immune cells from the stromal vascular fraction [37], as evidenced by the remarkable decrease in T cell marker *CD3E*, pan-macrophage marker *CD68*, endothelial cell marker *CD144* and antigen-presenting cell marker *HLA*-*DR*. In our post-obese group, *CASP3* expression in adipose tissue is even higher than in the obese group, clearly indicating a catabolic state.

TNF inhibits adipogenesis by downregulating *PPARG* expression in cultured human adipocytes [38]. We detected increased lipolysis and reduced adipogenesis in our cohort. The downregulation of metabolic markers *ADIPOQ*, *IRS2*, *PPARG*, and *SCL2A4* (GLUT-4) expression after weight loss indicates reduced metabolism in adipose tissue. A study performed in rats showed similar results after RYGB [39]. *PPARG* downregulation suggests an inhibition of adipogenesis and concomitant insulin resistance in SAT during weight loss [40]. Other studies also showed a decrease in *PPARG* after hypocaloric diet or 3 months after bariatric surgery [41–43]. Other adipogenesis-related genes such as *IGF1* [44] and *FGF1* were also significantly downregulated in the post-obese group. The preadipocyte differentiation marker *FGF1* was downregulated in post-obese SAT, contrary to the findings of Mejhert et al. who did not detect any differences in FGF1 secretion after 10 weeks of diet-induced weight loss [45]. However, the weight loss was, notably, not as pronounced as we observed 1 year after bariatric surgery.

Although insulin pathway genes were downregulated, whole-body insulin sensitivity was restored in the post-obese group as suggested by the HOMA-IR and as noted previously [21]. Surprisingly, *TNF* expression in post-obese could be predicted by fasting glucose, although fasting glucose values were already lower in comparison to the lean control group. Interestingly, *TNF* expression in SAT after bariatric surgery is independent of whole-body insulin resistance, contrary to the obese state. Local insulin resistance seems to occur in SAT, similar to cachectic states. In contrast to a previous study [46], the pro-inflammatory M1 macrophage marker *CD40* was not reduced in the post-obese group, although the pan-macrophage marker *CD68* expression was downregulated. The increased CD40/CD68 ratio together with the downregulation of *PPARG* and *ADIPOQ* suggest a shift towards local insulin resistance inducing M1-like macrophages in SAT [47–50]. M1 macrophages are the primary source of TNF and seem to orchestrate insulin resistance [51]. Interestingly, although the expression of the immune cell marker *CD40* does not change significantly, elastic net revealed *TNF* expression acts as a positive predictor. Therefore, CD40-positive cells could be a source of the *TNF* upregulation in the SAT of post-obese patients. Of note, an increased apoptosis rate may also cause *TNF* upregulation by the so-called apoptosis-induced apoptosis, where dying cells secrete TNF, which then in turn induce even more apoptosis [52]. A proposed mechanism for TNF modulation of lipolysis in adipose tissue after weight loss is described in Fig. 5.

Weight loss after bariatric surgery can sometimes lead to a pronounced reduction of fat and fat-free mass similar to cachexia [17]. Muscle wasting is relevant to cachexia; however, whole body muscle mass changes were not analyzed. Since myostatin (MSTN) levels correlate negatively with muscle mass and high circulating levels of MSTN were observed in muscle wasting conditions [53, 54], MSTN was used as surrogate marker of skeletal muscle mass. To our knowledge, we are the first to report changes in myostatin serum concentration after weight loss induced by bariatric surgery. Consistent with the findings of Park et al. in muscle biopsies of obese subjects ~1 year after bariatric surgery, we observed a significant decrease in circulating myostatin levels upon weight loss, which approximated the level of the lean group [55], suggesting that muscle wasting is not too advanced.

Regarding other growth factors related to both obesity and cachexia, our group showed that serum IGF-1 concentration increases in trend after surgery and is associated with a risk for developing post-load hypoglycemia 2 h after a standardized glucose challenge [21]. Downregulated *IGF1* in SAT in the post-obese group indicates that IGF-1-concentration is uncoupled from SAT expression, since serum IGF-1 is primarily hepatic [56].

Other examples of uncoupled SAT expression-serum concentration include circulating adiponectin and TNF. The increase in adiponectin concentration in serum 1 year after surgery did not reflect an increase in SAT *ADIPOQ* expression, although SAT is believed to be the main source of serum adiponectin [57], suggesting that in the post-obese, other fat depots, most likely bone marrow adipose tissue, significantly contributes to adiponectin serum levels [58]. Also, others observed that circulating adiponectin increases, while IL-6 and TNF concentration decreases upon weight loss [59, 60]. However, SAT *IL6* expression is described to be connected with serum levels in humans while *TNF* expression is not—meaning that TNF primarily acts in a paracrine or autocrine manner [61].

A limitation to our study is that the data merely present a “snapshot” during weight loss, as we did not include multiple time points after bariatric surgery. It would be interesting to know whether expression profiles after 5 or 10 years will resemble a healthy lean state in contrast to the catabolic state we observed in our study. An additional limitation of this study is that due to obvious reasons, we could only collect subcutaneous and not omental adipose tissue during follow-up. The amount of collected tissue by needle biopsy was very limited. Therefore, only mRNA expression could be investigated but neither histology nor protein expression. Adipose tissue samples were collected by excision during surgery for the lean and obese group and by needle biopsy in the post-obese group. Contradictory data are published on whether adipose tissue sampling affects gene expression analysis [77,78], yet we did not observe any significant differences between surgical excision and needle biopsy either (data not shown). For an inclusive statistical analysis, we performed a linear mixed effects model, taking into account the fact that due to sample size, considerations loss of power in comparing the lean with the obese group would occur.

Most studies focusing on bariatric surgery follow-up mainly investigated changes in serum parameters, whereas only few investigated changes in the expression of adipose tissue inflammation and cachexia-related genes. A major strength of our study is the thorough characterization of systemic and local adipose tissue changes after weight loss and the comparison to the lean state, as well as the novel finding that upon bariatric surgery, a state similar to cachexia seems to develop in SAT. Similarities and differences between post-bariatric weight loss as evaluated in this study and published data on cachexia [17, 62, 63] are presented in supplementary Table 2.

In summary, our data suggest that in response to surgery-induced weight loss, upregulated *TNF* expression leads to increased lipolysis via *PLIN1* downregulation, decreased adipogenesis via *PPARG* downregulation and even increased adipocyte turnover via *CASP3* upregulation. Concomitant with lipolysis, local insulin resistance also occurs in cachexia and was reflected in our study by the downregulation of *PLIN1*, *CIDEA*, *SLC2A4*, *ADIPOQ*, *IRS2*, and *PPARG*. Thus, the profound weight loss caused a specific pattern in SAT gene expression, which let us conclude that the post-obese SAT reflects a cachexia-like state rather than healthiness. For a more in-depth analysis of the biological processes occurring in different human fat depots during weight loss, the mechanisms through which these changes contribute to whole-body metabolism need to be investigated in further studies.

## Electronic supplementary material

Supplementary Table 1 (DOCX 17 kb)

Supplementary Table 2 (DOCX 18 kb)

Figure 5 (GIF 413 kb)

High Resolution Image (TIFF 579 kb)
