# Supplementary material for: Upregulated TNF Expression 1 Year After Bariatric Surgery Reflects a Cachexia-Like State in Subcutaneous Adipose Tissue
Source: Obes Surg. 2016 Nov 29;27(6):1514–23. doi: 10.1007/s11695-016-2477-5 (PMC5423994; doi:10.1007/s11695-016-2477-5)
Supplement: Supplementary file 1 — (DOCX 17 kb) [file 11695_2016_2477_MOESM1_ESM.docx]

**Supplementary Table 1 – List of TaqMan probes**

| Gene | Protein | Order No. |
| --- | --- | --- |
| ADIPOQ | Adiponectin | Hs00605917_m1 |
| CASP3 | Caspase-3 | Hs00234387_m1 |
| CCL3 | C-C motif chemokine 3 | Hs00234142_m1 |
| CD3E | T-cell surface glycoprotein CD3 epsilon chain | Hs99999153_m1 |
| CD40 | Tumor necrosis factor receptor superfamily member 5 | Hs00386848_m1 |
| CD68 | Macrosialin | Hs00199349_m1 |
| CDH5 | CD144, Cadherin-5 | Hs00901463_m1 |
| CIDEA | Cell death activator CIDE-A | Hs00154455_m1 |
| FGF1 | Fibroblast growth factor 1 | Hs00265254_m1 |
| HLA-DRA | HLA class II histocompatibility antigen, DR alpha chain | Hs00219575_m1 |
| IGF1 | Insulin-like growth factor 1 | Hs01547656_m1 |
| IL1B | Interleukin-1 beta | Hs00174097_m1 |
| IL6 | Interleukin-6 | Hs00985639_m1 |
| IL10 | Interleukin-10 | Hs00961622_m1 |
| IRS2 | Insulin receptor substrate 2 | Hs00275843_s1 |
| KRT18 | Keratin, type I cytoskeletal 18 | Hs02827483_g1 |
| PLIN1 | Perilipin-1 | Hs00160173_m1 |
| PPARG | Peroxisome proliferator-activated receptor gamma | Hs01115513_m1 |
| SLC2A4 | Solute carrier family 2, facilitated glucose transporter member 4 | Hs00168966_m1 |
| UBC | Polyubiquitin-C | Hs00824723_m1 |
| VEGFC | Vascular endothelial growth factor C | Hs00153458_m1 |
