# Supplementary material for: Upregulated TNF Expression 1 Year After Bariatric Surgery Reflects a Cachexia-Like State in Subcutaneous Adipose Tissue
Source: Obes Surg. 2016 Nov 29;27(6):1514–23. doi: 10.1007/s11695-016-2477-5 (PMC5423994; doi:10.1007/s11695-016-2477-5)
Supplement: Supplementary file 2 — (DOCX 18 kb) [file 11695_2016_2477_MOESM2_ESM.docx]

**Supplementary table 2. Proposed similarities and differences between cachexia and post-bariatric weight loss (WL)**

| Biological category | Post-bariatric WL | Cachexia |
| --- | --- | --- |
| Cytokines | ***TNF* ↑**  ***IL1B* ↓**  ***IL6* ↓** | ***TNF* ↑**  ***IL1B* ↑**  ***IL6* ↑** |
| Glucose metabolism /  Insulin sensitivity (IS) /  Insulin resistance (IR) | **systemic IS/local IR**  ***ADIPOQ* ↓**  ***SCL2A4* ↓** | **systemic IR***  ***ADIPOQ* ↓**  ***SCL2A4* ↓** |
| Lipolysis/Adipogenesis | ***PLIN1* ↓**  ***CIDEA* ↓**  ***PPARG* ↓** | ***PLIN1* ↓**  ***CIDEA* ↑**  ***PPARG* ↓** |
| Growth factors | **sIGF-1 ↑**  **sMyostatin ↓** | **sIGF-1 ↓**  **sMyostatin ↓** |
| Fat free mass | **↓**** | **↓** |
| Systemic inflammation | **↓** | **↑** |
| Muscle waisting | **not analyzed** | **↑** |

Differences in expression of genes prototypical for the indicated biological categories as observed in post-bariatric (this study) and cachectic patients (references below). sIGF-1 – IGF-1 serum concentration; sMyostatin – Myostatin serum concentration.

*M. Batista *et al.*, *J Endocrinol* **215**, 363–373 (2012), Y. Noguchi *et al.*, *Biochem. Biophys. Res. Commun.* **253**, 887–92 (1998). **C. Nicoletti *et al.*, *Nutrition (Burbank, Los Angeles County, Calif.)* **30**, 569–74 (2014).
