# Supplementary figures and images for: Upregulated TNF Expression 1 Year After Bariatric Surgery Reflects a Cachexia-Like State in Subcutaneous Adipose Tissue
Source: Obes Surg. 2016 Nov 29;27(6):1514–23. doi: 10.1007/s11695-016-2477-5 (PMC5423994; doi:10.1007/s11695-016-2477-5)

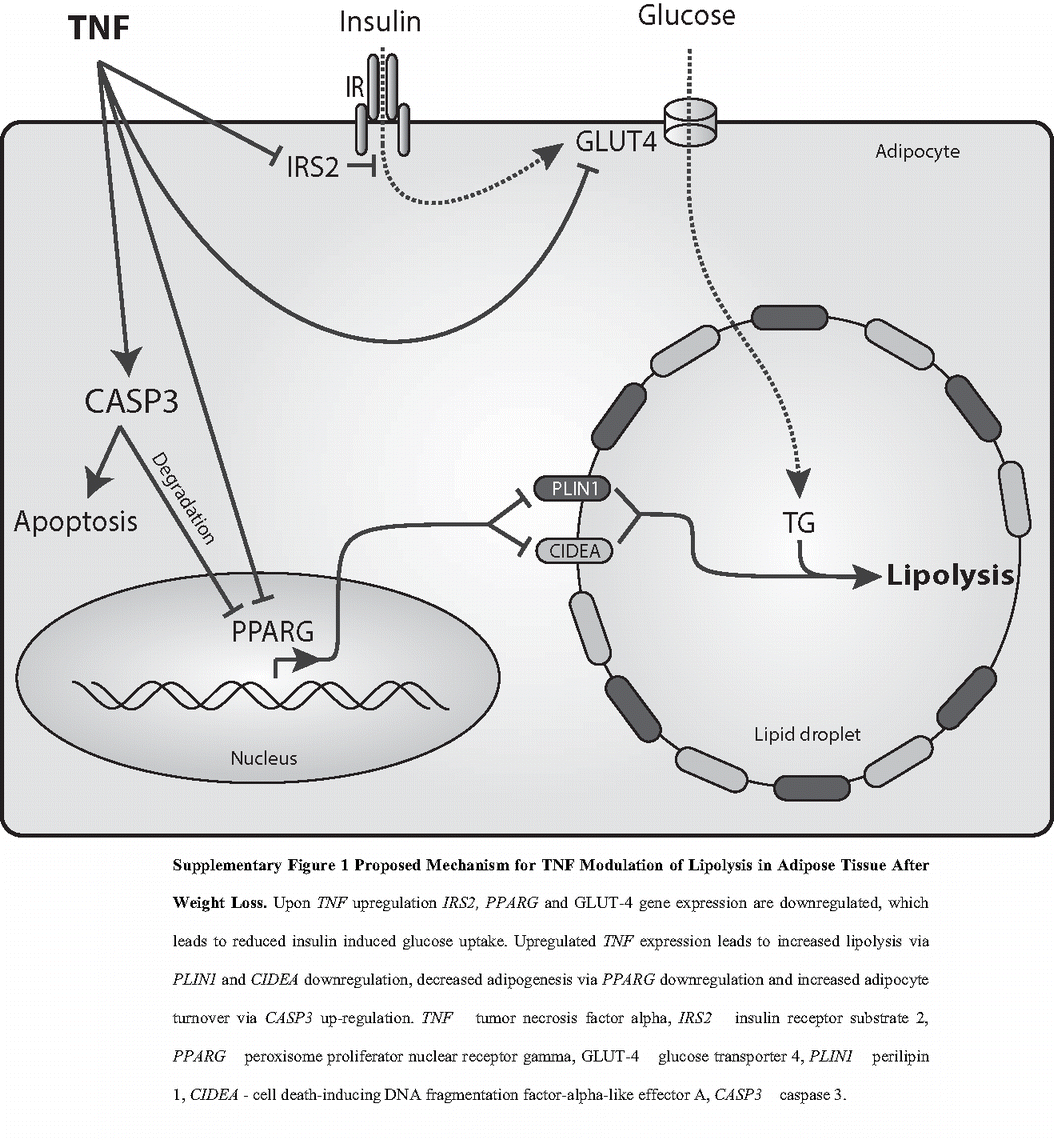

Supplement: Supplementary file 3 — (GIF 413 kb) [file 11695_2016_2477_Fig5_ESM.gif]

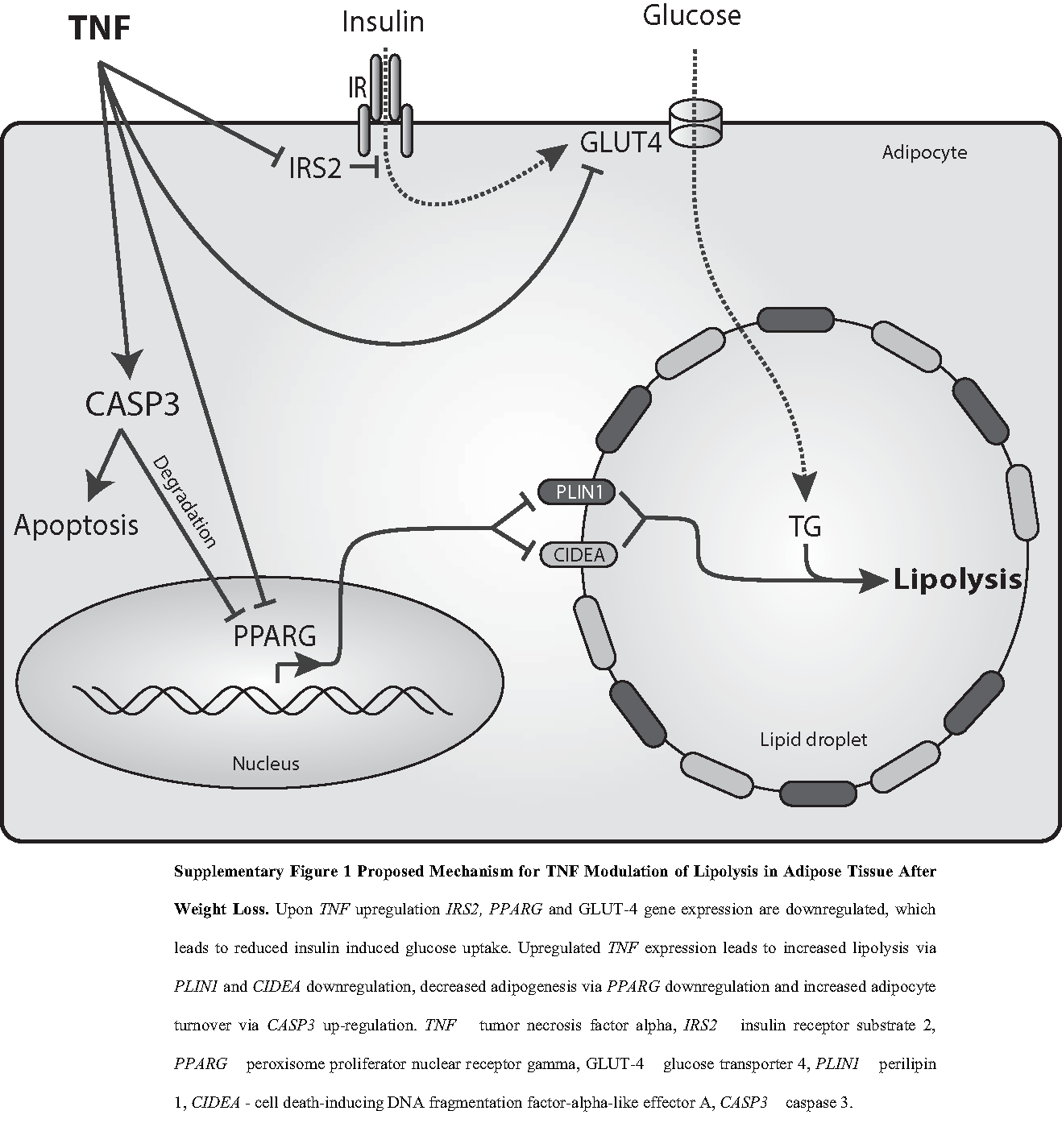

Supplement: Supplementary file 4 — High Resolution Image (TIFF 579 kb) [file 11695_2016_2477_MOESM3_ESM.tif]
